# Etiology and clinical features of children with bronchiectasis in China: A 10‐year multicenter retrospective study

**DOI:** 10.1111/crj.13630

**Published:** 2023-05-31

**Authors:** Hao Wang, Bao‐ping Xu, Yan‐min Bao, Yungang Yang, Li‐ling Qian, Hai‐lin Zhang, Chun‐mei Zhu, Yong Yin, Min Jiang, Ji‐hong Dai, Yong‐sheng Xu, Xiao‐hua Zhu, Xiao‐ping Zhu, Kun‐ling Shen

**Affiliations:** ^1^ Beijing Children's Hospital Capital Medical University Beijing China; ^2^ National Clinical Research Center for Respiratory Diseases Beijing China; ^3^ Shenzhen Children's Hospital Shenzhen Guangzhou China; ^4^ The first affiliated hospital of Xiamen University Xiamen Fujian China; ^5^ Children's Hospital of Fudan University Shanghai China; ^6^ The 2nd Affiliated Hospital and Yuying Children's Hospital of WMU Wenzhou Zhejiang China; ^7^ Capital Institute of Pediatrics Children's Hospital Beijing China; ^8^ Shanghai Children's Medical Center Shanghai China; ^9^ The First Affiliated Hospital of Guangxi Medical University Nanning Guangxi China; ^10^ Children's Hospital of Chongqing Medical University Chongqing China; ^11^ Tianjin Children's Hospital Tianjin China; ^12^ Jiangxi Provincial Children's Hospital Nanchang Jiangxi China; ^13^ The Affiliated Hospital of Guizhou Medical University Guiyang Guizhou China

**Keywords:** bronchiectasis, children, China, multicenter study

## Abstract

**Introduction:**

The current study aims to investigate the etiology spectrum and the clinical characteristics of bronchiectasis in Chinese children.

**Methods:**

The study is designed as a multicenter retrospective study. 193 cases were enrolled in 13 centers in China between 2008 and 2017. The inclusive cases must meet the clinical as well as the HRCT criteria. Only if both two radiologists confirmed the diagnosis, the case could be enrolled. The cases that could not provide clinical and imageology data were excluded. The data were entered into the specialized system and then analyzed.

**Results:**

One hundred sixty‐nine cases (87%) were found to have the underlying etiology. Post‐infective (46%), primary immunodeficiency (14%), and PCD (13%) were the common causes. All cases came from 28 provinces in Mainland China. The median age of symptom onset was 5.8 (2.0, 8.9) years. The median age of diagnosis was 8.4 (4.5, 11.6) years. The main symptoms were cough, sputum expectoration, and fever during the exacerbation. Nineteen percent of patients suffered from limited exercise tolerance. Clubbing was found in 17% of cases. Nearly 30% of patients presented growth limitations. On the HRCT findings, 126 cases had diffused bronchiectasis, and bilateral involvement was found in 94 cases. The lower lobes and right middle lobes were most commonly involved. Approximately 30% of cultures of sputum and bronchoalveolar lavage were positive.

**Conclusion:**

A majority of cases could be found the underlying etiology. Post‐infective, primary immunodeficiency, and PCD were the most common causes. Some clinical figures might indicate a specific etiology.

## Graphical abstract

Bronchiectasis patients usually suffer recurrent respiratory infections, and lung function is damaged progressively. It is not rare in China but is not well understood. Through the 10‐year retrospective multicenter study, we initially found the etiology spectrum and clinical characteristics of bronchiectasis in Chinese children.

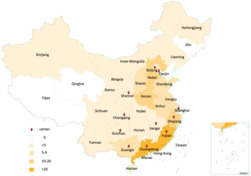

**Abbreviations**
BO: bronchiolitis obliterans
CF: cystic fibrosis
FEF_25–75_
: forced expiratory flow between 25% and 75% of FVC
FEV_1_
: forced expiratory volume in 1 s
FVC: forced vital capacity
HRCT: high‐resolution computed tomography
PCD: primary ciliary dyskinesia

## 1 INTRODUCTION

Bronchiectasis is morphologically defined as the permanent and abnormal dilation of the bronchi.^1^ The recurrent respiratory infection and progressive damage to lung function can influence the young patient's growth and quality of life negatively. Thanks to the emergence of high‐resolution computed tomography (HRCT) as the gold standard test, the diagnosis of bronchiectasis has become much easier. But the underlying etiology of bronchiectasis should still be a consideration for pedestrians. Bronchiectasis unrelated to cystic fibrosis (CF) is uncommon in children in Western countries, but it remains a problem in developing countries,^2^, ^3^, ^4^ and the composition of the etiology is more complex, which includes various respiratory infections, immunodeficiency, foreign body aspiration, primary ciliary dyskinesia (PCD), and so forth. The current study aims to investigate the etiology spectrum and the clinical characteristics of bronchiectasis in Chinese children. It is a multicenter study of children with bronchiectasis in China.

## 2 METHODS

### 2.1 Study subjects

**FIGURE 1:**
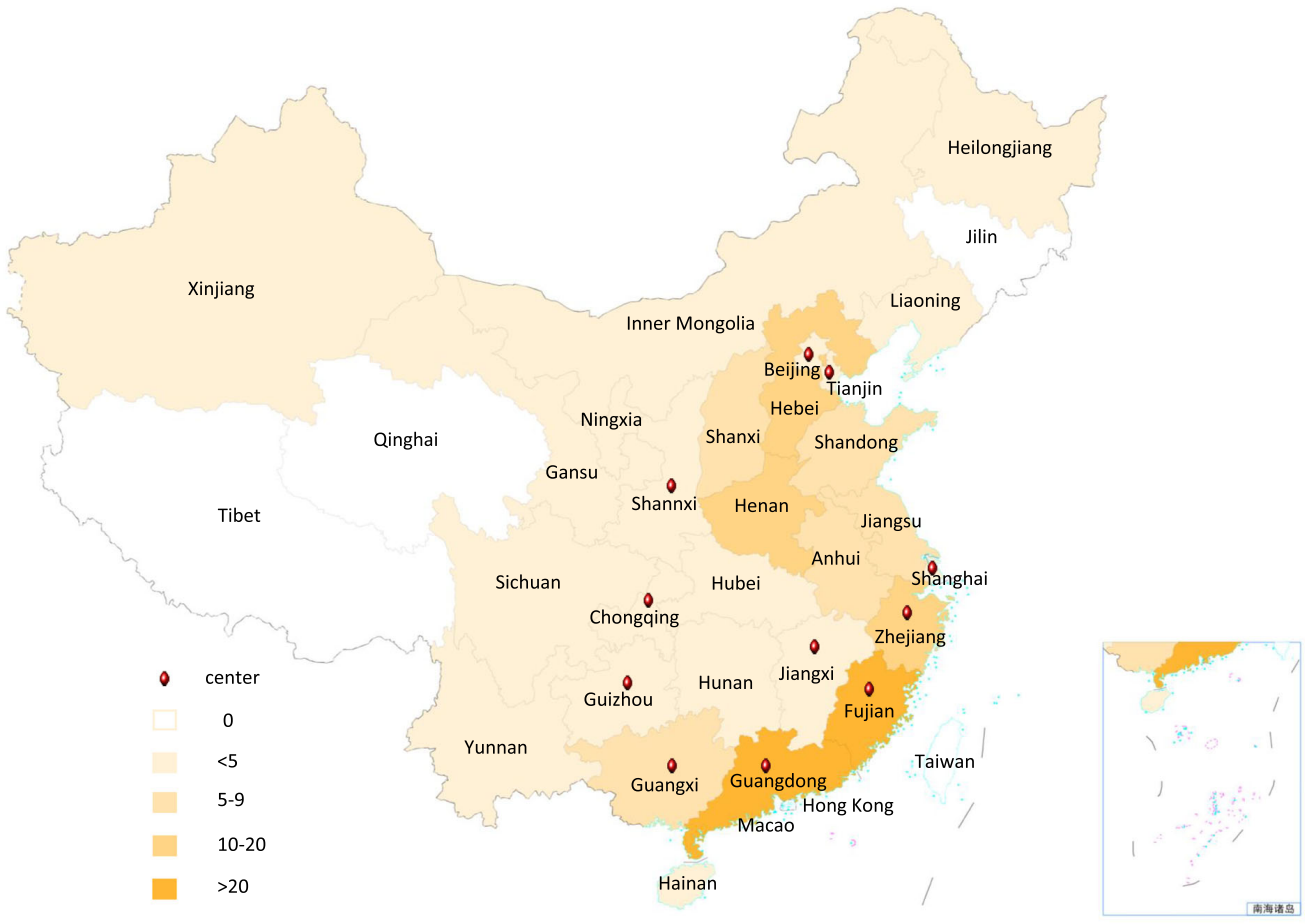
The distribution of bronchiectasis cases in China. This Chinese map showed the geographical distribution of the enrolled cases and the location (red point) of the current study centers. The different color bands demonstrated the range between the numbers of cases.

Two hundred twenty‐nine cases that were identified as having bronchiectasis by the electronic medical record system based on the International Classification of Disease‐10 between the beginning of 2008 and the end of 2017 were retrospectively screened in 13 centers. All 13 centers are pediatric pulmonary sections in tertiary‐care hospitals in 11 cities in China (Figure 1). According to the inclusion and exclusion criteria, 36 cases were excluded, and 193 cases were eventually enrolled.

### 2.2 Methods

The current study is designed as a multicenter retrospective study by reviewing the medical records with the standard detail chart, which got ethical approval from the Beijing Children's Hospital Research Ethics Board and did not need informed consent.

Firstly, all enrolled cases must be provided with HRCT image data to identify whether bronchiectasis can be diagnosed. Twenty‐nine cases were excluded because they could not provide the image data. Then, two pediatric radiologists evaluated all of the HRCT images independently and in a blinded fashion. The included case must coincide with at least one of the four items of HRCT manifestations: (1) internal bronchial diameter greater than the accompanying pulmonary artery; (2) lack of bronchial tapering; (3) bronchi visible in the peripheral 1 cm of the lung; and (4) bronchial diameter greater than adjacent segmental bronchi.^5^ Only if both radiologists confirmed the diagnosis could the case be enrolled. Three cases were excluded in this step. All of the associated clinical details and investigations were recorded on the predesigned standard forms. Four cases that could not provide important information such as demographic data, main clinical features, and etiology analysis were excluded. The clinical data of all‐inclusive cases were entered into OpenClinica Community version 3.4.1. The web‐based database was designed for this project by the Peking University Clinical Research Institute.

The collected data included date of birth, date of symptom onset, date of diagnosis, gender, permanent living address, family history, birth history, history of childhood respiratory infections, main symptoms, etiology‐associated symptoms, physical signs, imaging findings, spirometry, microbiology, and etiology analysis. Imaging findings included the chest X‐ray and HRCT at the first evaluation when diagnosed. The distribution in each lobe (regarding the lingual as a separate lobe) and the categorization (cylindrical or cystic) of bronchiectasis were identified. Bronchiectasis was defined as localized if only one lobe was affected and diffused if more than one lobe was found.^6^ Sixty‐three cases had spirometry records. Spirometry was performed in accordance with American Thoracic Society standards. The forced vital capacity (FVC) and forced expiratory flow between 25% and 75% of FVC (FEF_25–75_) measurements were expressed in percentage of the predicted value referred to the healthy population and the forced expiratory volume in 1 s/FVC ratio.

The underlying etiology of bronchiectasis was identified on the basis of the clinical manifestations and specific tests. And the patients were separated into eight groups by different etiologies. (1) Post‐infective: The post‐infective cause was considered when there was a history of previous severe pneumonia in the same lobe and the symptoms of bronchiectasis occurred after pneumonia. (2) Immunodeficiency: Tests such as immunoglobulin and lymphocyte subsets helped confirm the diagnosis of primary immunodeficiency. An HIV test and a history of immunosuppressive therapy were used to identify the acquired immunodeficiency. (3) Primary ciliary dyskinesia: The definitive diagnosis of PCD relied on transmission electron microscopy, which could find the typical defect in cilia structure or the gene test that showed the diagnostic pathogenic mutation. Kartagener syndrome was diagnosed when the patient had the triad of bronchiectasis, sinusitis, and situs inversus. If the patient had the suggestive clinical triggers of PCD but could not be diagnosed by transmission electron microscopy or gene testing, it was defined as suspicious PCD. (4) Foreign body: Patients with a previous history of foreign body aspiration and documentation by bronchoscopy could be diagnosed with this cause. (5) Gastric aspiration: 24 h‐esophageal pH monitoring and a barium swallow test were used to diagnose gastric aspiration cause. (6) Bronchiolitis obliterans (BO): Bronchiectasis could be one of the abnormalities found in BO. Repeated or continuous wheezing, coughing, and tachypnea, as well as mosaic ground‐glass patterns on HRCT, were the characteristic features. (7) Other causes: Other causes, such as Steven–Johnson syndrome, SLE, and so forth, could be diagnosed depending on the characteristic history and investigations. CF is rare in China, so it is not excluded from this study. A sweat test and gene test helped confirm the diagnosis. (8) Unknown: Patients who did not have a suggestive trigger on clinical figures or investigations were considered to have an unknown cause.

### 2.3 Statistical analysis

IBM SPSS Statistics version 24.0 software was used for data analysis. Categorical variables were compared by the chi‐square test or Fisher's exact test (when the expected cases were less than five); the ANOVA test was used to compare normally distributed continuous variables, which were presented as mean ± standard deviation. The nonparametric test was used to compare non‐normally distributed continuous variables, which were presented as median (IQR) and range. Values of *P* ≤ 0.05 were considered significant.

## 3 RESULTS

### 3.1 Etiology

A total of 169 cases (86.6%) were found to have the underlying etiology. Post‐infective was the most common cause in the present study. Primary immunodeficiency and PCD were the most common congenital causes. There were still many other causes found (Table 1).

**TABLE 1:** The etiology composition of bronchiectasis.

Etiology	Number of cases	% (*n* = 193)
Post‐infective	89	46.1
Mycoplasma	26	
Measles	3	
Adenovirus	1	
Tuberculosis	5	
*Streptococcus pneumonia*	6	
Other pathogens	6	
Mixed infection	16	
Unknown pathogen	26	
Immunodeficiency	26	13.5
primary immunodeficiency	25	
XLA	14	
CVID	3	
Other primary immunodeficiency	8	
AIDS	1	
PCD	25	13.0
Definite	12	
Suspicious	13	
Foreign body	7	3.6
Gastric aspiration	6	3.1
BO	5	2.6
Others Congenital defects of airways or lung	11 3	5.7
Interstitial lung disease	3	
CF	2	
SLE	1	
Bronchial artery pulmonary artery fistula	1	
Steven–Johnson syndrome	1	
Unknown	24	12.4

Abbreviations: AIDS, acquired immune deficiency syndrome; BO, bronchiolitis obliterans; CF, cystic fibrosis; CVID, common variable immunodeficiency disease; PCD, primary ciliary dyskinesia; SLE, systemic lupus erythematosus; XLA, X‐linked agammaglobulinemia.

### 3.2 Demographic data and clinical characteristics

The total of 193 eligible cases came from 28 provinces, autonomous regions, and municipalities directly under the central government of Mainland China (Figure 1). The majority of cases came from Guangzhou (28 cases), Fujian (27 cases), Henan (19 cases), Hebei (17 cases), and Zhejiang (16 cases).

**FIGURE 2:**
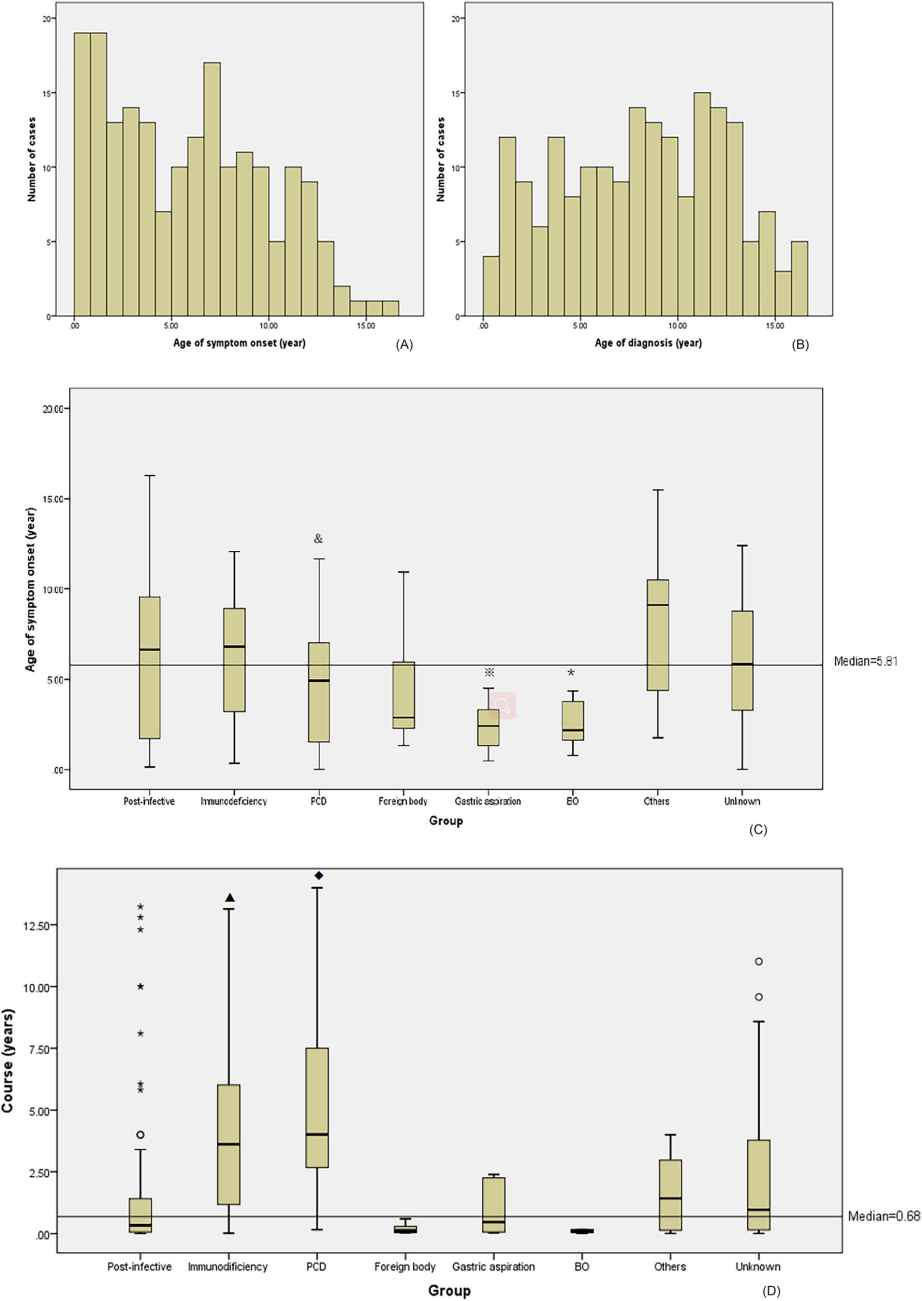
The demographic features of bronchiectasis. (A) The distribution of age of symptom onset. (B) The distribution of age of diagnosis. (C) The age of symptom onset in different etiology groups. The horizontal line represented the median age of symptom onset in all cases. The additional character represented the statistically significant difference between the two groups. (D) The course between symptom onset and diagnosis in different etiology groups. The horizontal line represented the median course in all cases. & The symptom onset age of the primary ciliary dyskinesia (PCD) group (median = 4.9 years) was statistically significantly lower than that of the other causes group (median = 9.1 years) (*P* = 0.047); ※ the symptom onset age of the gastric aspiration group (median = 2.4 years) was statistically significantly lower than the immunodeficiency group (median = 6.8 years) (*P* = 0.008), the other causes group (median = 9.1 years) (*P* = 0.021), and the unknown group (median = 5.9 years) (*P* = 0.034); * the symptom onset age of the bronchiolitis obliterans (BO) group (median = 2.2 years) was statistically significantly lower compared with the immunodeficiency group (median = 6.8 years) (*P* = 0.015) and other causes (median = 9.1 years) group (*P* = 0.020); ▲ the course of the immunodeficiency group (median = 3.6 years) was statistically significantly longer than every other etiology group (*P* < 0.05) except PCD; ◆ the course of the PCD group (median = 4.0 years) was statistically significantly longer than every other etiology group (*P* < 0.05) except immunodeficiency.

The median age of symptom onset was 5.8 (2.0, 8.9) years (range, 1 week to 16 years and 3 months). The median age of the first diagnosis of bronchiectasis was 8.4 (4.5, 11.6) years (range, 6 months to 16 years and 7 months). The median course between symptom onset and diagnosis was 8.2 (1.5, 42.3) months (range, 1 month to 14 years). There was a statistically significant difference by median test (*P* = 0.022) to compare the median age of symptom onset and clinical course between the etiology groups (Figure 2). There were 118 males and 74 females (1.6:1).

The main symptoms and signs were shown in Table 2; in addition, 27.9% and 27.6% of patient's height and weight percentiles were under the 3rd percentile on the standardized growth charts for the same gender and age, respectively.^7^ One hundred percent of PCD patients presented sputum expectoration, which was significantly higher than the post‐infective (65.2%) (*P* < 0.001), foreign body (42.9%) (*P* = 0.001), and BO (20.0%) (*P* < 0.001) groups. Immunodeficiency patients presented more recurrent respiratory infections (69.2%) compared with the post‐infective (34.8%) (*P* = 0.003), foreign body (0.0%) (*P* = 0.002), and unknown (25.0%) (*P* = 0.002) groups. PCD patients also presented significantly more clubbing (48.0%) compared with the post‐infective group (6.7%) (*P* < 0.001). There wasn't a statistically significant difference in any other symptoms or signs between the etiology groups.

**TABLE 2:** The main symptoms and physical signs of bronchiectasis.

Symptoms and signs	Number of cases	% (*n* = 193)
Cough	188	97.4
Sputum expectoration	144	74.6
Fever during the exacerbation	124	64.2
Recurrent respiratory infection	82	42.5
Wheeze	60	31.1
Hemoptysis^a^	14	7.3
Limited exercise tolerance^b^	37	19.2
Abnormal lung auscultation Crackles Wheeze Phlegm Mixed	108 64 18 59 52	56.0 33.2 9.3 30.6 26.9
Clubbing	32	16.6
Dyspnea	24	12.4

^a^ Seven cases presented blood‐streaked sputum, and the hemoptysis volume of the other seven patients was less than 10 mL per day.

^b^ Thirty‐seven point eight percent of exercise‐limited cases could not maintain a normal life and dropped out of school.

### 3.3 Imaging and lung function

The chest X‐ray results were detected in 123 cases. Only 21 (17.1%) of them could find bronchiectasis on a chest X‐ray. Sixty cases (48.8%) showed pneumonia features. Forty‐two cases (34.1%) just demonstrated the increase and disorder of the lung markings. On the HRCT finding (Table 3), there were totally 450 lobes involved in all the patients. Besides bronchiectasis, there were some other abnormal findings in the patients. Infiltration in the pulmonary parenchyma and interstitial was found in 116 cases and 34 cases, respectively. Thirty‐one patients performed atelectasis. A mucus plug was found in 11 cases. To compare the HRCT findings between the etiology groups, there wasn't a significant difference in the distribution and categorization of bronchiectasis.

**TABLE 3:** HRCT findings of bronchiectasis.

Etiology	LU	L	LL	RU	RM	RL	Diffused (% of each etiology)	Bilateral (% of each etiology)	Cystic (% of each etiology)
Post‐infective	15	21	46	18	50	55	59 (58.1)	38 (42.7)	25 (28.1)
Immunodeficiency	5	6	20	5	18	16	18 (69.2)	16 (61.5)	7 (26.9)
PCD	4	9	15	6	20	14	20 (80.0)^a^	18 (72.0)^b^	5 (20.0)
Foreign body	1	0	3	0	0	4	1 (14.3)^a^	0 (0)^b^	5 (71.4)
Gastric aspiration	1	2	2	2	2	3	5 (83.3)	2 (33.3)	3 (50.0)
BO	0	2	3	2	4	5	5 (100)	4 (80.0)	1 (20.0)
Others	3	6	4	3	6	6	7 (45.8)	6 (54.5)	2 (18.2)
Unknown	4	5	12	4	10	8	11 (65.3)	10 (41.7)	12 (50.0)
Total	33	51	105	40	110	111	126 (65.3)	94 (48.7)	60 (31.1)

*Note*: Each cell showed the number of involved lobes or cases.

Abbreviations: L, lingular lobe; LL, left lower lobe; LU, left upper lobe; RL, right lower lobe; RM, right middle lobe; RU, right upper lobe.

^a^ There was a statistically significant difference in the diffused cases ratio between the PCD group and the foreign body group (*P* = 0.003).

^b^ There was a statistically significant difference in the bilateral involved cases ratio between the PCD group and the foreign body group (*P* = 0.001).

The mean FVC%, FEV_1_/FVC%, and FEF_25–75_% were 74.8 ± 18.1%, 87.8 ± 12.7%, and 70.9 ± 29.9%, respectively. There was a statistically significant difference in FEV_1_/FVC% between the post‐infective and PCD groups (95.6 ± 11.1% vs. 83.1 ± 13.5%, *P* = 0.03).

### 3.4 Bronchoscopy and microbiology

All 146 bronchoscopy results showed endobronchial inflammation. Bronchiectasis was detected by bronchoscopy in 27 cases. Forty‐three cases performed a large number of viscous secretions obstructing the bronchial, and 64% of PCD cases had this performance, which was an extremely high ratio compared with others (*P* < 0.001). The stenosis of the bronchi was found in 13 cases.

Microbiology findings are shown in Table 4. In the sputum *Pseudomonas aeruginosa* positive cases, there were three PCD, two post‐infective, and one CF. Two PCD patients and one CF patient had positive *P. aeruginosa* cultures in bronchoalveolar lavage and sputum.

**TABLE 4:** Microbiology of sputum and bronchoalveolar lavage culture.

Pathogen	Sputum (*n* = 95) (number of cases)	Bronchoalveolar lavage (*n* = 64) (number of cases)
*S. pneumonia*	6	4
*Haemophilus influenza*	6	2
*P. aeruginosa*	6	3
Others	8	5
Mixed infection	4	4
Fungus	2	2
Total positive cases	32	20
Positive rate (%)	33.7	31.3

## 4 DISCUSSION

The etiology of non‐CF bronchiectasis is complicated. Identifying the underlying etiology is more important than just diagnosing bronchiectasis because this may alter management.^8^ The systemic review of previous data showed that 63% of children with non‐CF bronchiectasis had an underlying etiology, and previous pneumonia (17%), primary immunodeficiency (16%), aspiration (including foreign body and recurrent aspiration) (10%), and PCD (9%) were implicated most commonly.^9^ In the present study, 86.6% of cases identified the underlying and common causes, which is similar to the previous analysis. Bronchiectasis is one of the sequels of severe pneumonia. The most common etiology in this group was post‐infection (46.1%), which was much higher than the systemic review data. The previous studies in China reported that the proportion of post‐infective was 30%–68%,^2^, ^10^ which might be because pneumonia was still a big problem in China. The incidence of measles and tuberculosis decreased rapidly in the past decades, so only about 5% of post‐infective bronchiectasis cases were sequenced by measles or tuberculosis in this study, whereas one‐third were mycoplasma. Mycoplasma is one of the most important pathogens in children with community‐acquired pneumonia in China, and the incidence is increasing in these years. Mycoplasma infection damaged the airway epithelium directly by active infection, indirectly by infection‐induced immune mechanisms, or both,^11^ which could be a trigger of a “vicious cycle” leading to progressive destruction of bronchial walls resulting in dilatation and airflow obstruction.^12^ Whereas some previous studies reported the improvement or reversibility of post‐infection bronchiectasis patients,^2^, ^13^ reasonable therapy and long‐term follow‐up are essential for this kind of patient. In the present study, primary immunodeficiency (13.0%) and PCD (13.0%) were the most common congenital causes that were similar to the previous study.^9^ Hypogammaglobulinemia was the major form of immunodeficiency associated with bronchiectasis. Only half of the PCD patients were definitely diagnosed by electron microscopy. For one thing, some PCD patients' ciliary function was abnormal but with normal substructure^14^; for another, in some cases, infection caused secondary damage to the cilia. So, for the suspicious cases, the combination of ciliary substructure, ciliary function, nasal nitric oxide, and gene testing was essential and to avoid getting an epithelium sample during the infection. A long‐term follow‐up also helps make a definite diagnosis.

The median age of symptom onset was 5.8 (2.0, 8.9) years in this group, the distribution of which was skewed (Figure 2A). Forty‐four percent of the patients presented symptoms before the age of 5 years old. So clinicians should be aware of the possibility of bronchiectasis, even in young children. The PCD, gastric aspiration, and BO patients were tending to have earlier onset (Figure 2C), so for the early symptom onset age cases, these above etiologies should be taken into consideration. Although the majority (55.6%) of the patients could be diagnosed in 1 year, there were still some cases of delayed diagnosis, even by more than 10 years. It was reported that patients with unknown etiologies had a longer duration.^15^ In this study, the course of the immunodeficiency and PCD groups was statistically significantly longer than every other etiology group, which reminds the clinician that immunodeficiency and PCD might be easy to misdiagnose.

Chronic productive cough and abnormal lung auscultation were the most common symptoms and signs in this group, as in previous reports.^2^, ^16^ Wheeze was not rare in children with bronchiectasis. In this group, nearly one‐third of patients presented with wheezing, and 71.7% of them had the symptom onset before 5 years old. Hemoptysis can be an alarming and life‐threatening complication of bronchiectasis, and it is not uncommon in adult patients (37%),^17^ whereas it is relatively rare in the children's group.^2^, ^3^ There were 7.3% of cases that presented hemoptysis in this group, and there wasn't persistent or significant hemoptysis reported. The previous data reported that the incidence of clubbing in pediatric bronchiectasis patients was variable, about 4%–35%.^3^, ^18^ In this study, 16.6% of cases presented clubbing. It was reported that height and body mass index were related to the severity of the disease and the delay in diagnosis.^19^ The limitation of growth and exercise tolerance was not uncommon in this group, which demonstrated that bronchiectasis indeed affects growth and quality of life. Although we could not identify the etiology just by the symptoms and signs, and most of them did not have a relation to etiology in the present study, there are still some features demonstrated more commonly in a certain cause. All PCD patients presented with sputum expectoration. Clubbing could be found in nearly half of the PCD patients. Immunodeficiency patients presented with more recurrent respiratory infections. If the bronchiectasis patients have the manifestations above, the clinician should take PCD or immunodeficiency into consideration.

Thanks to the advent of HRCT, the diagnosis of bronchiectasis has become easier, and it is now considered the gold standard for diagnosing bronchiectasis.^12^, ^20^ Probably because of gravity, mucociliary clearance is more efficient in the upper lobe, so bronchiectasis is easier to occur in the lower lobe.^20^ In the present study, the most commonly involved lobes, in turn, were the right lower lobe (24.7%), right middle (24.4%) lobe, and left lower lobe (23.3%), similar to the previous report.^16^ There wasn't any statistically significant difference in distribution comparing etiology, so we could not identify the etiology by the distribution of bronchiectasis lesion on HRCT.^8^ Although cystic bronchiectasis usually denotes a longstanding and more severe disease, the usefulness of categorizing bronchiectasis into cylindrical, varicose, or cystic subtypes is limited.^21^ In this study, cylindrical bronchiectasis was found in the majority of cases, whereas there wasn't any relationship between the etiology and categorizing bronchiectasis. The categorization of bronchiectasis could not help distinguish the etiology. Although the statistically significant difference in diffused and bilaterally involved cases ratio was only found between the PCD group and the foreign body group, half of the immunodeficiency, PCD, and BO patients demonstrated diffused and bilateral involvement in HRCT (Table 3). These causes should be taken into consideration when meeting extensive bronchiectasis cases. The Australian study showed that patients with or without immunodeficiency had little effect on lung function.^19^ In the present study, it was found that PCD patients had lower FEV_1_/FVC% compared with the post‐infective group, whereas there was no other difference between etiologies. The following prospective study is needed to focus on the variation of lung function over time.

Bronchoscopy is an important method to detect and manage foreign bodies that can cause bronchiectasis.^21^ In the present study, all seven foreign body cases were diagnosed by bronchoscopy. Except that bronchoscopy can also help obtain the bronchoalveolar lavage sample and the bronchial epithelium sample for the diagnosis of PCD. We also found that most PCD cases performed viscous secretions obstructing the bronchi, which was an extremely high ratio compared with others. That might be an indication of PCD. In this study, *S. pneumonia* was the most common pathogen found in sputum and bronchoalveolar lavage. Most of the *P. aeruginosa* was found in the PCD and CF cases. Mixed infections were not rare.

The limitation of this study was that it was a retrospective study, and the clinical data were collected from the hospital‐based electronic medical record system, so some information was incomplete. A prospective study and longtime follow‐up cohort were needed for the following work.

## 5 CONCLUSION

It was a multicenter study of children with bronchiectasis in China. A majority of cases could be found the underlying etiology. Post‐infective was the most common cause, whereas primary immunodeficiency and PCD were the most common congenital causes. Although the etiology should be definite by specific examinations, some clinical characteristics can help the clinician find the tendency of such causes.

## AUTHOR CONTRIBUTIONS

Hao Wang designed the study, collected data, carried out the initial analyses, drafted the initial manuscript, and reviewed and revised the manuscript. Bao‐ping Xu and Kun‐ling Shen conceptualized and designed the study, coordinated and supervised data collection, and critically reviewed the manuscript for important intellectual content. Yan‐min Bao, Yungang Yang, Li‐ling Qian, Hai‐lin Zhang, Chun‐mei Zhu, Yong Yin, Min Jiang, Ji‐hong Dai, Yong‐sheng Xu, Xiao‐hua Zhu, and Xiao‐ping Zhu participated in and approved the design of the study, recruited the eligibility cases from each center, collected and managed data, and reviewed and revised the manuscript. All authors approved the final manuscript as submitted and agreed to be accountable for all aspects of the work.

## CONFLICT OF INTEREST STATEMENT

None.

## ETHICS STATEMENT

The current study got ethical approval from the Beijing Children's Hospital Research Ethics Board and did not need informed consent. The ethics number is 2015‐81.

## Data Availability

The data that support the findings of this study are available from the corresponding author upon reasonable request.
